# Essential Role of the m_2_R-RGS6-I_KACh_ Pathway in Controlling Intrinsic Heart Rate Variability

**DOI:** 10.1371/journal.pone.0076973

**Published:** 2013-10-29

**Authors:** Ekaterina Posokhova, David Ng, Aaisha Opel, Ikuo Masuho, Andrew Tinker, Leslie G. Biesecker, Kevin Wickman, Kirill A. Martemyanov

**Affiliations:** 1 Department of Neuroscience, The Scripps Research Institute, Jupiter, Florida, United States of America; 2 Genetic Diseases Research Branch, National Human Genome Research Institute, Bethesda, Maryland, United States of America; 3 Department of Medicine, University College London, London, United Kingdom; 4 The Heart Centre, William Harvey Research Institute, Barts and the London School of Medicine and Dentistry, Charterhouse Square, London, United Kingdom; 5 Department of Pharmacology, University of Minnesota, Minneapolis, Minnesota, United States of America; Cardiological Center, Italy

## Abstract

Normal heart function requires generation of a regular rhythm by sinoatrial pacemaker cells and the alteration of this spontaneous heart rate by the autonomic input to match physiological demand. However, the molecular mechanisms that ensure consistent periodicity of cardiac contractions and fine tuning of this process by autonomic system are not completely understood.

Here we examined the contribution of the m_2_R-I_KACh_ intracellular signaling pathway, which mediates the negative chronotropic effect of parasympathetic stimulation, to the regulation of the cardiac pacemaking rhythm. Using isolated heart preparations and single-cell recordings we show that the m_2_R-I_KACh_ signaling pathway controls the excitability and firing pattern of the sinoatrial cardiomyocytes and determines variability of cardiac rhythm in a manner independent from the autonomic input. Ablation of the major regulator of this pathway, Rgs6, in mice results in irregular cardiac rhythmicity and increases susceptibility to atrial fibrillation. We further identify several human subjects with variants in the *RGS6* gene and show that the loss of function in RGS6 correlates with increased heart rate variability. These findings identify the essential role of the m_2_R-I_KACh_ signaling pathway in the regulation of cardiac sinus rhythm and implicate RGS6 in arrhythmia pathogenesis.

## Introduction

Regular contractions of the heart are essential for its normal function. Disruption in the rhythmicity of this process manifests in cardiac arrhythmias, a broad group of diseases that are among key risk factors for developing stroke, heart failure, and sudden cardiac arrest [1]. The primary role in generating and maintaining cardiac rhythm belongs to the sinoatrial pacemaking cells where activity of several ion channels drives spontaneous and periodic contractions [2].

There is a considerable variability in the intervals between consecutive heartbeats *in vivo*, known as sinus arrhythmia or heart rate variability (HRV). It is a physiological phenomenon that is attributed, in large part, to dynamic changes in autonomic input to the heart [3]. Parasympathetic activation of post-ganglionic neurons innervating the heart leads to a decrease in heart rate (HR) and makes heartbeat less regular, which is reflected by an increase in HRV [4], [5]. A positive correlation of parasympathetic tone to HRV, combined with observations that parasympathetic stimulation tends to counteract the pro-arrhythmic effects of sympathetic activation led to use of HRV as an independent predictor of morbidity and mortality associated with myocardial infarction, congestive heart failure, and congenital heart disease in humans [6], [7].

Parasympathetic stimulation results in the release of acetylcholine that binds to the m_2_ subtype of muscarinic receptor (m_2_R) on atrial pacemaking cells, triggering activation of pertussis-toxin sensitive, inhibitory heterotrimeric G proteins leading to the reduction in cellular excitability. The K^+^ channel I_KACh_ plays a central role in this process, accounting for at least half of the inhibitory response caused by the m_2_R stimulation [8]. The channel is composed of two subunits, Girk1 and Girk4, which form an obligate heterotetramer gated by direct association with activated G proteins [9]. Several recent studies indicated that the duration of G protein signaling from the m_2_R to the I_KACh_ plays a critical role in determining the extent of the parasympathetic inhibition of the heart rate [10], [11], [12], [13], [14]. The time that G proteins spend in the activated state is controlled by the Regulator of G protein Signaling (RGS) proteins that serve as negative regulators of m2R-I_KACh_ signaling [15], [16]. Accordingly, rendering G proteins insensitive to RGS action [10], [11] or eliminating specific RGS proteins (*i.e.*, Rgs4 [12] or Rgs6 [13], [14]) exacerbates the bradycardic response and/or increases an occurrence of heart block upon m_2_R activation. While the function of RGS in controlling effects of parasympathetic inhibition of the HR is well established, their role in regulating cardiac rhythmicity is largely unexplored.

In this study we used isolated heart preparation to dissect contributions of m_2_R-I_KACh_ signaling pathway in controlling the regularity of cardiac contractions as measured by the HRV. We show that a key regulator of this pathway, Rgs6, plays an essential role in curbing sinus arrhythmia in a manner independent from parasympathetic activation. Loss of Rgs6 in mice and disruption of orthologous gene product in humans lead to increased HRV and susceptibility to atrial fibrillation.

## Materials and Methods

### Antibodies, Recombinant Proteins, DNA Constructs

All general chemicals and atropine were purchased from Sigma Aldrich (St. Louis, MO). Carbachol was from Acros Organics (Geel, Belgium). The cDNA construct encoding wild-type human *RGS6* was obtained from Missouri S&T cDNA Resource Center. Single point mutations were generated by Mutagenex Inc. (Piscataway, NJ) and were verified by sequencing.

### Mouse Strains

The generation of *Rgs6^−/−^* [14] and *Girk4^−/−^* [17] mice has been described previously. Mice were out-bred onto the C57BL/6 background for at least 5 generations unless stated otherwise. All procedures were carried out in accordance with the National Institute of Health guidelines and were granted formal approval by the Institutional Animal Care and Use Committees of the University of Minnesota and The Scripps Research Institute.

### Telemetry

4–5 month old mice were used for *in vivo* ECG monitoring as described [14]. ECG signals were digitized at 1 kHz sampling rate, exported as text files, and analyzed for HRV as described below.

### Langendorff isolated heart preparation and drug administration

Mice (8–12 wks) were heparinized (100 IU) and anesthetized using isoflurane (Halocarbon, River Edge, NJ). Hearts were rapidly excised and immediately cannulated for retrograde aortic perfusion in a constant pressure mode (60 mmHg) with modified Krebs-Henseleit buffer containing (in mM): 118.5 NaCl, 25 NaHCO_3_, 4.7 KCl, 1.2 KH_2_PO_4_, 11 D-glucose, 1.2 MgSO_4_, 1.8 CaCl_2_, 2 sodium pyruvate. The buffer solution was filtered (0.22 µm) and saturated with 95% O_2_–5% CO_2_ at 38°C. Hearts were allowed to stabilize for 30 min, and were excluded from pharmacological experiments and HRV analysis if any of the following was present: (i) persistent arrhythmia >5 min, (ii) HR below 250 bpm, (iii) stable steady-state HR not attained within the first 20 min. Hearts with signs of ischemia upon dismounting from the apparatus were also excluded. Drugs were added to the perfusate and various concentrations were applied in a cumulative manner (7–8 min each). Atropine was administered at 1 µM concentration.

### Langendorff heart rate data analysis

*Ex vivo* data were acquired using the PowerLab data acquisition system (ADInstruments, Colorado Springs, CO) and digitized at a sampling rate of 2 kHz. LabChart Pro v.7 software with HRV and dose-response plug-ins (ADInstruments, Colorado Springs, CO) was used for all data analysis. Additional channels were set up for “cyclic measurements” to convert raw ECG data into beat-to-beat HR using default mouse ECG settings and “smoothing” to calculate 10-s moving averages of beat-to-beat HR.

Heart rate dynamics of all hearts without signs of ischemia were visually evaluated during the stabilization period (0–30 min) and the following parameters were calculated: (i) presence of persistent arrhythmia episodes (>5 min), (ii) presence of persistent bradycardic episodes (10-s average heart rates of <250 bpm for >5 min), (iii) number of bradycardic episodes (10-s average heart rates <250), (iv) number of sinus arrhythmia episodes (sinus rhythm; >15% change in HR between two consecutive 10-s averages). Basal heart rates were quantified within a 10 min window using HRV plug-in of LabChart Pro v7 as described below. Non-linear fitting of dose-response data and EC_50_ analysis was done in GraphPad Prism5 using least squares fitting method.

### HRV analysis

*Ex vivo* data were acquired using PowerLab data acquisition system (ADInstruments, Colorado Springs, CO) and digitized at a sampling rate of 2 kHz. LabChart Pro v.7 software with HRV and dose-response plug-ins (ADInstruments, Colorado Springs, CO) was used for all data analysis. For HRV analysis, a “maximum after threshold” algorithm was used for R peak detection. Noisy data segments and ectopic beats were manually excluded from analysis. Signal preprocessing, threshold and retrigger delay values were altered when necessary to ensure all the peaks within the selected window were labeled correctly. All HRV parameters were analyzed in the 5 min interval preceding drug treatment for isolated hearts, or over a 5 min total of appended consecutive intervals of telemetry recording (baseline), or within the last 5 min window of drug application for isolated hearts. For time domain analysis, the following parameters were calculated: mean normal-to-normal interval (NN, ms), standard deviation of all NN intervals (SDNN, ms), and square root of the mean square of successive differences between adjacent NN intervals (RMSSD, ms), number and percentage of consecutive NN intervals differing by over 50 ms (NN50 and pNN50, correspondently; were not observed *ex vivo*). Frequency domain analysis was done with FFT (Fast Fourier Transformation) size of 1024 and a Welch window with half overlap. Frequency bands were defined as follows: 0.4–1.5 Hz, low frequency (LF); 1.5–5 Hz, high frequency (HF). Power in each of the bands and total power (TP, 0.0–10 Hz; ms^2^) were calculated. LF and HF were also expressed in normalized units (nu; (LF or HF)*100/(TP-VLF)), and LF/HF ratio was determined.

### SAN cells isolation from adult mice

SAN cells were isolated from adult mice (6–8 wks) as described [14] and used within 8 h of isolation.

### Calcium imaging and data analysis

All calcium imaging experiments were performed on acutely-dissociated SAN cells within 6 h of plating. SAN cells were loaded with the cell-permeable 2 mM Indo-1/AM (Life Technologies) for 20 min in modified KB media followed by incubation in KB media for at least 20 min prior to recordings. Immediately prior to the experiment, media was exchanged to HEPES-Hanks media containing (in mM): 20 HEPES, 137 NaCl, 1.3 CaCl_2_, 0.4 MgSO_4_, 0.5 MgCl_2_, 5.4 KCl, 0.4 KH_2_PO_4_, 0.3 Na_2_HPO_4_, 3 NaHCO_3_, 5.6 glucose. Coverslips were mounted in a perfusion chamber and positioned on a movable stage of a Leica CTR6000 inverted microscope. Indo-1 was excited using 380 nm UV light using a dichroic filter cube and a 40× objective (Leica). Emitted light was then passed through a DV2 dual-view image splitter (MAG Biosystems) equipped with band-pass filters of 405 nm (30 nm cutoff) and 485 nm (25 nm cutoff). 405 nm and 485 nm images were acquired using a Hamamatsu Em CCD camera as a stream of 1078 frames with 15 ms exposure time and 1 ms timelapse. Ratiometric images of Indo-1 fluorescence were analyzed by defining regions of interest outlining the cell soma using MetaFluor Software version 7.6.1.0 (MDS Analytical Technologies).

Acquired data were exported, and 16 s recordings were analyzed for oscillation frequency (F) and RMSSD according to the following formulae:
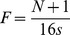
(1)where N is total number of peak-to-peak intervals (RR) within the recording
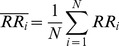
(2)

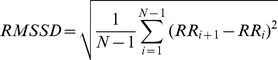
(3)Peak detection for RR interval calculation was performed using peak analyzer feature of OriginPro 8.6 software (OriginLab Corp., Northampton, MA). Skipped beats were defined as RR intervals with over 15% difference in duration with the interval immediately before or after it.

### Whole-cell electrophysiology

Coverslips containing SAN cells were transferred to a perfusion chamber and electrophysiological recordings were conducted as previously described [14]. Only those experiments for which the access resistances were stable and low (<15 MΩ) were included in the final analysis.

### In-vivo electrical pacing

*In vivo* electrophysiological studies using the EPR-800 catheter were carried out as previously described [18]. Atrial burst pacing was found to be the most consistent method for AF induction, and was performed at a rate of 600 bpm (100 ms coupling interval) for 25 s after which pacing was switched off. The rate was subsequently increased in 10 ms increments, the minimum coupling interval being 10 ms. Both atrial burst pacing and programmed electrical stimulation with the addition of atrial extras was used to induce AF in the *Rgs6^−/−^* and wild-type mice. This was through the addition of atrial extra-stimuli (‘extras’) following a train of 8 beats at rates of 100 to 10 ms; single to 10 extras were inserted at coupling intervals from 75 to 10 ms. Carbachol (0.5 mg/kg i.p.) was injected to decrease heart rate by at least 20% before burst pacing was repeated. An episode of AF was defined as an irregular rhythm with a normal QRS complex and no discernible P waves lasting longer than 1 s. The arrhythmia was reproducible when it occurred and had to be induced on more than one occasion under identical conditions.

### Human patients and exome genotyping

Patient recruitment, clinical evaluation, and exome analysis was performed as described [19], [20]. Mutation descriptions correspond to Human Genome Variation Society standards and are referenced to GenBank NM_004296.5. The ClinSeq study was prospectively reviewed and approved by the NHGRI IRB. This approval included written informed consent for acquisition, analysis, and publication of unidentified data.

### Heart rate variability analyses of human subjects

ECG was recorded continuously for 24 h using EVO Holter monitoring system (Spacelabs Healthcare, Issaquah, WA). Initial Holter data editing and peak labeling was performed by a qualified physician in a semi-automatic mode using Cadionavigator+ Impresario Holter Analysis System V.3.07.0158 (Spacelabs Heathcare, 2001). HRV analysis was then performed by a blinded investigator. For each Holter recording, one 15 min epoch was selected at the beginning of each hour during the day (8:00–18:00) and night (23:00–06:00) periods. Each epoch was then manually examined for correct labeling of the peaks and Delmar Reynolds/Centum HRV analyzer (2003; Delmar Reynolds Medical) was used for the analysis. The Fast Fourier Transformation (FFT) method was used for frequency domain analysis. Frequency and time domain analysis was carried out separately for each epoch and was then averaged within each Holter recording either for all epochs (total), or separately for daytime and nighttime.

### Analysis of RGS activity by the BRET assay

Agonist-dependent cellular measurements of bioluminescence resonance energy transfer (BRET) between masGRK3ct-Nluc and Gβ1γ2-Venus were performed to visualize the action of G protein signaling in living cells as previously described [21], [22]. To generate masGRK3ct-Nluc reporter, amino acids G495-L688 of bovine GRK3 (NP_776925), preceded by a myristic acid attachment peptide (mas; MGSSKSKTSNS) were fused with the NanoLuc [23] via the GGGS linker. The average baseline value recorded prior to agonist stimulation was subtracted from BRET signal values, and the resulting difference was normalized against the maximal value recorded upon agonist stimulation. The rate constants (1/μ) of the deactivation phases were obtained by fitting a single exponential curve to the traces. *k*_GAP_ rate constants were determined by subtracting the basal deactivation rate (*k*_app_) from the deactivation rate measured in the presence of exogenous RGS protein.

### Statistical Analysis

All data are reported as mean ± SEM. Statistical analyses were performed using Prism (GraphPad Software, Inc.; La Jolla, CA) software. EC_50_ values were calculated with the Hill coefficient set to 1. The impact of atropine on HRV response was evaluated using paired Student's t-test, while the unpaired t-test was used elsewhere. Whenever equal variance criteria were not met, logarithmic transformation (log2) was applied to the data prior to using Student's t-test, where possible. Otherwise, the Student's t-test with Welch correction was used. For all analyses, the level of significance was set at *P*<0.05. Electrophysiological parameters were evaluated using one-way ANOVA or students t-test (two-tailed), as appropriate. Tukey's HSD *post hoc* test was used for pairwise comparisons when genotype was found to exert an influence on an electrophysiological parameter. For the analysis of the BRET assay kinetic data non-parametric Kruskal-Wallis One Way Analysis was used.

## Results

### m2R-I_KACh_ pathway sets heart rate variability independently from the autonomic input

**Figure 1:**
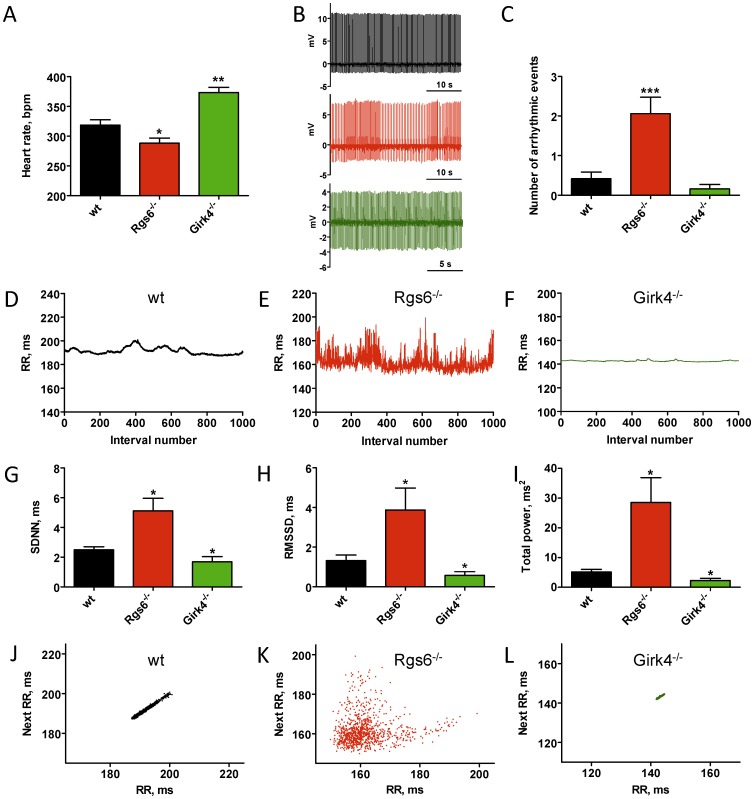
Rgs6 and Girk4 have opposite effects on HRV in isolated hearts. A, Average HR in hearts isolated from wild-type (wt, n = 36), *Rgs6* ^−/−^ (n = 52), and *Girk4* ^−/−^ (n = 19) mice. B, ECG traces recorded in isolated wild-type (black), *Rgs6^−/−^* (red), and *Girk4^−/−^* (green) hearts. Note rhythm irregularity in *Rgs6* ^−/−^ hearts. C, Quantification of sinoatrial dysfunction events. D–F, Representative tachograms of baseline ECG in wild-type (black), *Rgs6* ^−/−^ (red), and *Girk4* ^−/−^ (green) hearts. G–I, Key HRV parameters in the time and frequency domains from ECG recordings. J–L, Non-linear HRV analysis by Poincare plots for wild-type (J), *Rgs6^−/−^* (K), and *Girk4^−/−^* (L) hearts. Symbols: * P<0.05, ** P<0.01, ***P<0.001 vs. wild-type.

To investigate the role of m_2_R-I_KACh_ signaling in maintaining regularity of cardiac contractions, we evaluated mice with a gain (*Rgs6^−/−^*) or a loss (*Girk4^−/−^*) of pathway function. We performed these studies in isolated hearts to distinguish intrinsic cardiac properties from changes triggered by the autonomic nervous system. Analysis of the HR indicated that *Rgs6^−/−^* hearts showed significant bradycardia, while the loss of I_KACh_ in *Girk4^−/−^* hearts led to a significant tachycardia (Fig. 1A), suggesting that these molecules affect cardiac properties independently from autonomic input.

Remarkably, *Rgs6^−/−^* hearts showed signs of sinus node dysfunction indicated by episodes of abrupt changes in sinus rhythm (Fig. 1B). These episodes were rare in wild-type and *Girk4^−/−^* hearts but occurred with high frequency in *Rgs6^−/−^* hearts (Fig. 1C). We next performed quantitative analysis of cardiac rhythm regularity by evaluating HRV, a measure that reports the periodicity of cardiac contractions by sampling variations in the time interval between heartbeats [24]. Representative tachograms (Fig. 1D–F, Fig. S1 in File S1) showed a prominent decrease in the consistency of beat-to-beat intervals in *Rgs6^−/−^* hearts relative to wild-type controls. At the same time, rhythm fluctuations in *Girk4^−/−^* hearts were virtually eliminated. Consistent with these observations, several quantitative parameters of HRV in both time and frequency domains, were significantly increased in *Rgs6^−/−^* hearts and decreased in *Girk4^−/−^* hearts (Fig. 1G–I and Table S1 in File S1). Furthermore, non-linear analysis of HRV using the Poincare method [25], [26] also showed a significant difference in rhythm regularity between the genotypes (Fig. 1 J–L; Fig. S2 in File S1). Indeed, the linear-type dispersion pattern common to wild-type hearts was reduced to a dot-like profile with minimal degree of scatter in *Girk4^−/−^* hearts. In contrast, disruption of *Rgs6* substantially increased plot dispersion. Because there was no significant difference in HR among ECG segments used for HRV analysis, we concluded that the observed changes in variability did not stem from the effects on overall HR. Taken together, these data suggest that regulation of I_KACh_ by an Rgs6-controlled intrinsic cardiac signaling pathway is essential for ensuring the regularity of sinus rhythm.

### Rgs6 regulates cardiac automaticity by inhibiting I_KACh_ activity in SAN cells

**Figure 2:**
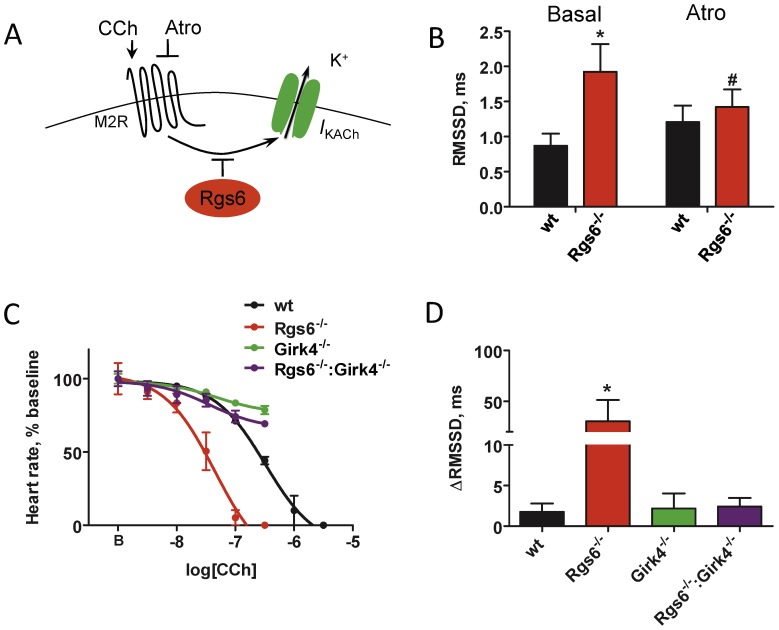
The effects of the Rgs6 on HRV are mediated by the I_KACh_ and are influenced by the m_2_R activity. A, Schematic representation of the pathway targeted both genetically and pharmacologically. Abbreviations are: atropine (Atro), carbamylcholine (CCh). B, Effect of m_2_R blockade by atropine on HRV in wild-type (black; n = 7) and *Rgs6^−/−^* hearts (red; n = 10). No significant effect of drug was observed in wild-type hearts. C, Increased sensitivity of *Rgs6^−/−^* hearts to m_2_R stimulation and its rescue by I_KACh_ (*Girk4*) ablation. Increasing concentrations of CCh were applied to isolated perfused hearts (n = 4–6 per genotype). D, m_2_R stimulation non-proportionately increased HRV in *Rgs6^−/−^* hearts. Hearts (n = 3–6 per genotype) were perfused with CCh (∼IC_10_ concentration) identified from dose-response studies, followed by measurement of changes in the RMSSD parameters. Symbols: * P<0.05 vs wild-type, #P<0.05 vs treatment.

To determine the extent of I_KACh_ contribution to the dysregulation of sinus rhythm in *Rgs6^−/−^* hearts, we generated a *Rgs6^−/−^:Girk4^−/−^* double knockout mouse line. Concurrent ablation of *Girk4* and *Rgs6* completely abolished the effect of Rgs6 loss on HRV, bringing it to values observed in hearts from *Girk4^−/−^* mice (Table S1 in File S1), arguing that I_KACh_ is the primary mediator of Rgs6 influence on HRV. Because G protein activation is required for opening of the I_KACh_ channel and because Rgs6 is a negative regulator of G proteins, we reasoned that increased I_KACh_ activity in isolated *Rgs6^−/−^* hearts results from an increased level of spontaneous G protein activation (Fig. 2A). Indeed, application of atropine, an m_2_R antagonist with inverse agonist properties [27], significantly decreased HRV in hearts from *Rgs6^−/−^* mice without affecting HRV in wild-type hearts (Fig. 2B).

We hypothesized that stimulation of m_2_R with carbamylcholine (CCh) would have an opposite effect from atropine, and disproportionately increase cardiac variability in isolated *Rgs6^−/−^* hearts. Indeed, dose-response studies showed about 5 times greater sensitivity of *Rgs6^−/−^* hearts to the negative chronotropic effects of CCh relative to hearts from wild-type mice (IC_50_: 125±51 nM vs. 504±63 nM; Fig. 2C). Conversely, in *Girk4^−/−^* hearts, CCh was relatively ineffective, reducing HR by less than 20% at the maximal concentration used (Fig. 2C). Increased sensitivity to m_2_R stimulation in *Rgs6^−/−^* hearts was eliminated by the concurrent disruption of *Girk4*, indicating that I_KACh_ is the sole mediator of the increased sensitivity to m_2_R stimulation seen in *Rgs6^−/−^* mice. We also compared HRV across the genotypes at equivalent minimal doses of CCh that insignificantly altered HR (∼IC_10_: 3 nM for *Rgs6^−/−^*, and 30 nM for other genotypes). While m_2_R stimulation mildly increased HRV across all genotypes (Fig. 2D), *Rgs6^−/−^* hearts responded with a dramatic increase in HRV, supporting the hypothesis that increased I_KACh_ activity in the absence of Rgs6 is driven by disinhibition of m_2_R signaling.

**Figure 3:**
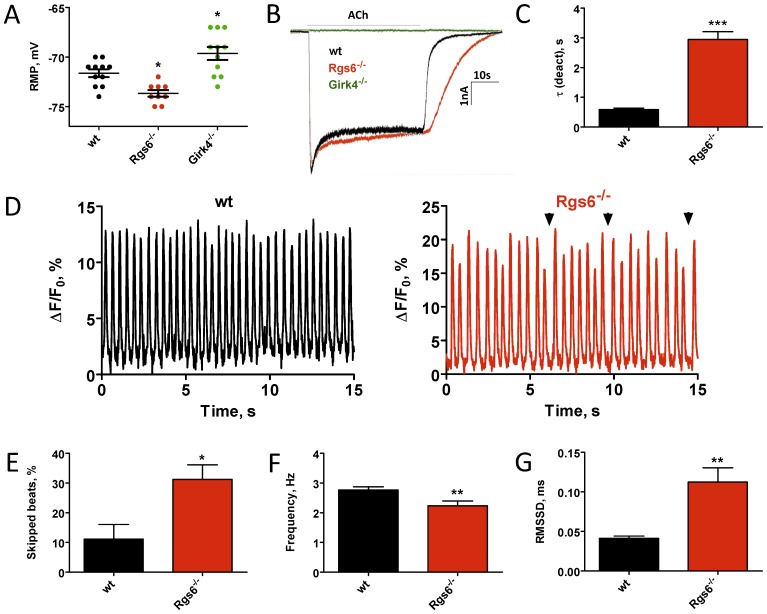
Ablation of *Rgs6* reduces excitability of sinoatrial cells and disrupts their automaticity. A, Resting membrane potential measured immediately after obtaining whole-cell access in wild-type (wt), *Rgs6^−/−^*, and *Girk4^−/−^* SAN cells. B, Inward currents evoked by application of acetylcholine (ACh, 100 µM) in SAN cells from wild-type (black), *Rgs6^−/−^* (red) and *Girk4^−/−^* (green, no current) mice. C, Summary of steady-state ACh-induced deactivation kinetics of I_KACh_ in wild-type and *Rgs6^−/−^* SAN cells (n = 11–15 cells/genotype). D, Representative traces of spontaneous calcium oscillations recorded from wild-type (black; n = 14) and *Rgs6^−/−^* (red, n = 20) SAN cells. Arrows show skipped beats. E, Quantification of SAN arrhythmic events defined as more than 15% change in duration of peak-to-peak interval of spontaneous calcium oscillations in wild-type (n = 11) and *Rgs6^−/−^* (n = 17) SAN cells. F, Reduced frequency of spontaneous calcium oscillations recorded in *Rgs6^−/−^* SAN cardiomyocytes as compared to wild-type (n = 14–20 cells/genotype). G, Increased variability of spontaneous calcium oscillations in *Rgs6^−/−^* SAN cells as determined by increase in RMSSD values (n = 14–20 cells per genotype). Symbols: *P<0.05; **P<0.01; ***P<0.001.

We next analyzed how hyperactivity of the I_KACh_ channel affects responses at the single cell level. Electrophysiological recordings from isolated sinoatrial nodal (SAN) cells showed that resting membrane potential in *Rgs6^−/−^* cells was significantly more hyperpolarized than in wild-type SAN cells. Conversely, *Girk4^−/−^* SAN cells had significantly more depolarized resting membrane potentials (Fig. 3A). Application of acetylcholine (ACh) induced robust I_KACh_ currents in SAN cells from wild-type and *Rgs6* ^−/−^ mice, but not in cells from *Girk4* ^−/−^ mice (Fig. 3B and Table S2 in File S1). Consistent with previous observations [13], [14], it took substantially longer for the currents in *Rgs6* ^−/−^ SAN cells to deactivate upon agonist removal, indicating that Rgs6 ablation substantially augmented the ability of the I_KACh_ to remain open (Fig. 3C). Next, we analyzed how the observed decrease of cellular excitability in *Rgs6^−/−^* SAN cells affects the periodicity of spontaneous action potential generation in SAN cells. Calcium transients recorded from *Rgs6^−/−^* SAN cells were markedly irregular (Fig. 3D; Movie S1 in File S2) and displayed a significantly higher number of delayed firing events (Fig. 3D,E) as compared to wild-type SAN cells. This resulted in a substantial decrease in the frequency of spontaneous transients (Fig. 3F) and an increase in the variability of pacemaking activity (Fig. 3G).

### Disruption of Rgs6 increases HRV in mice and humans

**Figure 4:**
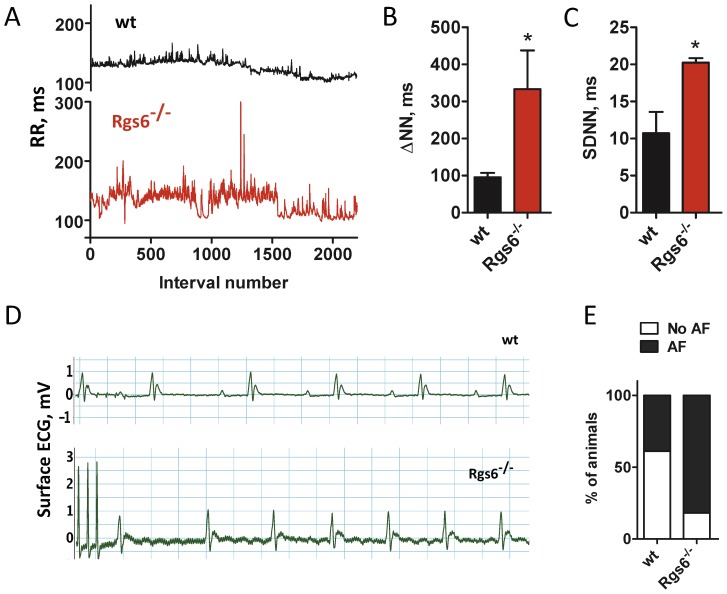
Inactivation of *Rgs6* disrupts cardiac rhythm in mice. A, Representative tachograms of RR intervals from wild-type (black) and *Rgs6^−/−^* (red) mice recorded by ECG radiotelemetry. B and C, Summary of HRV analysis in conscious, freely-moving mice. D, Burst pacing induced AF in *Rgs6^−/−^* but not in wild-type mice. Note an irregular rhythm with no discernible P waves in the *Rgs6^−/−^* recording. E, Quantification of AF induction probability. Symbols: *, P<0.05.

Having established the molecular mechanism by which Rgs6 contributes to automaticity, we next sought to determine the impact of its ablation on cardiac physiology *in vivo*. Analysis of ECG radiotelemetry recordings from conscious freely-moving *Rgs6^−/−^* mice showed a significant disruption of sinus rhythm (Fig. 4 A–D), as indicated by the observed increase in many HRV parameters (Fig. 4 C,D; Table S3 in File S1). We next asked whether increased HRV in *Rgs6^−/−^* mice predisposes or protects mice to/from atrial fibrillation (AF). Although no spontaneous AF was found in *Rgs6^−/−^* mice, burst pacing and/or programmed electrical stimulation of the heart resulted in the increased number of AF episodes in *Rgs6^−/−^* mice relative to wild type littermates (Fig. 4E,F; 82% vs 39%, *P* = 0.04). These episodes were characterized by a markedly irregular rhythm with a normal QRS complex and no discernible P waves.

**Figure 5:**
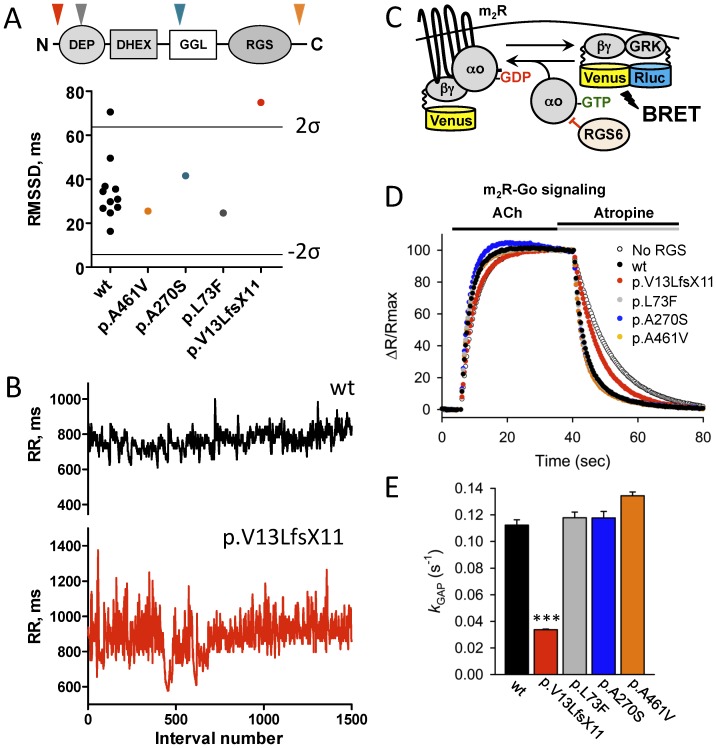
Abnormal sinus arrhythmia in a human subject with dysfunctional RGS6. A, HRV measured in humans carrying variants in *RGS6* and 11 age-matched control subjects (wt, black). Lines represent upper (2σ) and lower (−2 σ) 95% confidence thresholds as determined by the 2σ rule. *Insert*: domain structure of RGS6 protein. Arrows show localization of corresponding variants. B, Representative tachograms of RR intervals from a control subject (black) and a subject heterozygous for the p.Val13LeufsX11 variant in the *RGS6* gene (red) determined from continuous Holter recordings. C, Schematics of the assay design to study effects of mutations on the RGS6 function. Stimulation of the m_2_R by ACh results in the dissociation of Gμo from the heterotrimer. Released Gβγ subunits tagged with Venus become available for the interaction with Nluc8-tagged GRK reporter producing the BRET signal. D. Representative responses to sequential application of ACh (10 µM) and atropine (1 mM) recorded in the presence of the indicated constructs. The BRET signals averaged from 4 experiments were plotted as individual data points. *E*, Catalytic activity of RGS6 defined by the *k*_GAP_ parameter. To determine the *k*_GAP_ values, the deactivation rate constant measured in the absence of RGS6 was subtracted from values measured in the presence of RGS6. Symbols: ***, p<0.001 (n = 4).

To evaluate the relevance of these findings to human cardiac physiology, we identified one frame-shift and several non-synonymous variants in the coding region of human *RGS6* by exome sequencing [20]. All of the variants introduced amino acid changes in different domains of the RGS6 protein (Fig. 5A). Four subjects were heterozygous for a variant in *RGS6*: c.37del; p.Val13LeufsX11, c.217C>T; p.Leu73Phe, c.808G>T; p.Ala270Ser, or c.1382C>T; p.Ala461Val were evaluated in parallel with 11 age-matched control subjects with no mutations in *RGS6* sequence with 24 hour Holter monitors. Overall, Holter ECG recordings of all the subjects appeared normal with episodes of sinus tachycardia and/or sinus bradycardia. No conduction or rhythm abnormalities were detected. None of the *RGS6* variant carriers differed significantly from the control group with respect to minimum, maximum, and mean (total, daytime, and nighttime) HR.

Analysis of HRV showed a significant scatter of the HRV parameters in the control group (Fig. 5A, Table S4 in File S1). To assess the significance of the genotype effect, we set a 95% confidence threshold for deviation from the mean value of the control population. With these criteria, only the individual carrying the p.Val13LeufsX11 *RGS6* variant exhibited increased irregularity of the sinus rhythm outside of the normal range (Fig. 5A,B). In fact, out of the 31 parameters of HRV in both time and frequency domains that were measured, 14 were significantly increased in this individual (Table S4 in File S1). The p.Val13LeufsX11 variant is a single nucleotide deletion that predicts a frame-shift and a premature stop codon. In contrast, variants in *RGS6* predicting missense changes (p.Ala461Val, p.Ala270Ser and p.Leu73Phe) did not significantly affect HRV (Fig. 5A, Table S4 in File S1).

We next assessed the effects of these four variants on RGS6 function. We used a bioluminescence resonance energy transfer (BRET) based cellular assay to evaluate the ability of RGS6 to promote G protein inactivation, a biochemical activity of RGS proteins responsible for their regulatory effects on the GPCR signaling. In this assay, wild-type RGS6 promoted rapid Gμo deactivation following termination of m2R signaling by atropine. Consistent with the clinical data, the p.Ala461Val, p.Ala270Ser and p.Leu73Phe variants did not affect the ability of RGS6 to regulate m_2_R signaling (Fig. 5C,D). In contrast, introduction of the p.Val13LeufsX11 variant severely disrupted the ability of RGS6 to regulate m2R signaling. These data suggest that disruption of RGS6 function may be responsible for the increase in HRV in this individual.

## Discussion

Collectively, our findings reveal a role of the m_2_R-Rgs6-I_KACh_ pathway in controlling cardiac pacemaking activity. While previous studies established critical role of RGS proteins in regulating changes in the heart rate in response to parasympathetic activation, our current results implicate Rgs6 as a major determinant of the cardiac rhythmicity based on the analysis of the heart rate variability. These observations, together with reports of sinus arrhythmia in denervated hearts [28], [29], [30], reinforce the idea that the variation of spontaneous cardiac rhythm, or HRV, is independent from the autonomic input. Indeed, our data suggest that spontaneous ligand-independent activity of m_2_R drives I_KACh_ opening to suppress SAN excitability, thereby preventing generation of the regular sinus rhythm. By adjusting I_KACh_ gating, the key regulator of this pathway (Rgs6) sets the excitability threshold for consistent generation of action potentials by SAN cells. Elimination of Rgs6 increases I_KACh_ activity, resulting in hyperpolarization of SAN pacemaker cells. This, in turn, increases fluctuation in timing between individual depolarization events, manifesting as elevated HRV. In this conceptual framework, parasympathetic stimulation simply taps into this intrinsic m_2_R-Rgs6-I_KACh_ mechanism that controls regularity of the rhythm, augmenting sinus arrhythmia in addition to reducing HR.

It is interesting to compare our results with other studies reporting molecular perturbations that affect sinus rhythm. Indeed, more negative maximum diastolic potential of SAN cells lacking HCN2 [31] or HCN4 channels [32], resulted in the sinus dysrhythmia. Inhibiting HCN channels with cilobradine produced a similar effect [33]. Interestingly, sinus node arrhythmia was also observed in Ca_v_1.3^−/−^ SAN cells [34], and in isolated hearts upon blockade of the voltage-sensitive Na_v_1.1/1.3 sodium channels [35]. These channels are considered as primary molecular determinants of cardiac pacemaking mechanism. However, it is becoming accepted that rhythmic action potential generation requires collaborative action of several ion channels that form a “membrane voltage clock” [36]. Our data, together with evidence implicating GIRK4 in the pathogenesis of long-QT syndrome [37] reinforce the idea that I_KACh_ is another component of this intrinsic clock.

In contrast to other pacemaking channels that are actively engaged in action potential generation/oscillations, I_KACh_ plays more passive, gate-keeping role. Its tonic inhibition by Rgs6 is required to maintain normal sinus node function. Thus, in case of the I_KACh_, role in controlling the rhythm is delegated to the intracellular regulator, an RGS protein. Signaling by GPCRs, is well known to adjust the function of ion channels regulating the “membrane voltage clock”, creating flexibility in pacemaking rhythm [36]. One of the most prominent molecular mechanisms of such regulation in SAN cells involves changes in cAMP production that impacts the function of many ion channels [2]. For example, activation of m_2_R, in addition to opening I_KACh_, results in reduction in cAMP concentration which would inhibit HCN and Ca_v_1.3 channels [2]. Furthermore, the bradycardiac effects of parasympathetic activation were found to be only partially dependent on I_KACh_ [36]. Since Rgs6 facilitates termination of Gμi/o-dependent signaling [38], it could be expected to change cAMP homeostasis and thereby influence activity of the cAMP-sensitive pacemaking channels. However, our data indicate that elimination of *Girk4* in mice lacking Rgs6 completely rescues the increased HRV phenotype, arguing that dysregulation of I_KACh_ is the major source of sinus rhythm variation in this mouse model.

It is possible that the role of in sinus arrhythmia via modulation of I_KACh_ function is not restricted to Rgs6. Indeed, another member of RGS family, Rgs4, has been shown to regulate the m_2_R-I_KACh_ signaling pathway in a manner similar to Rgs6 [12], [39]. Given the SAN-specific expression pattern of Rgs4 [12], the two RGS proteins may play synergistic roles in setting the rhythm of cardiac automaticity and predispose to the development of disorders associated with increased parasympathetic input to the heart, including bradycardia, sinus node dysfunction, and atrial fibrillation [16]. However, the contribution of Rgs4 to these processes has yet to be determined.

Increased HRV is considered indicative of the prevalent parasympathetic tone, and as such, is used as a positive prognostic factor for predicting cardiovascular survival rates [7], [40]. As an integral parameter, HRV *in vivo* does not discriminate between changes in autonomic input from changes in the intrinsic excitability of cardiac tissue. The results of this study illustrate that the molecular dysfunction in an intrinsic pacemaking mechanism rather than changes in autonomic inputs could be a primary cause of sinus dysrhythmia and heart rate variability change. Differences in the molecular components that regulate sinus rhythm generation may explain heritability of HRV [41] and may also serve as a predisposition factors to AF. Indeed, the observed increase in HRV in hearts from *Rgs6^−/−^* mice was accompanied by an increased susceptibility to AF *in vivo*. This is consistent with the anti-arrhythmic effects of both genetic [42] and pharmacological I_KACh_ ablation [43], [44], as well as with the presence of constitutively-active I_KACh_ in animals with chronic AF [45] and in human patients with AF [46].

Although its role in the development of AF in human patients remains to be established, RGS6 function is clearly important for maintaining of regular cardiac rhythm in humans. This may illustrate the benefits of genetic screening for variants in m_2_R-RGS6-I_KACh_ pathway in an effort to reduce the risks of inducing dysrhythmia and/or severe bradycardia, when exposed to pharmacological treatments that elevate parasympathetic tone. Finally, this study suggests that the m_2_R-RGS6-I_KACh_ signaling pathway is a potential target for anti-arrhythmic pharmacological intervention.

## Supporting Information

File S1 **File includes Table S1–S4 and Figures S1–S2.** Table S1. Heart rate variability parameters in isolated hearts of wild-type, *Rgs6^−/−^*, *Girk4^−/−^*, and *Rgs6^−/−^:Girk4^−/−^* double knockout mice. Hearts were isolated from 10–12 mice (8–16 weeks old) per genotype. Abbreviations: HRV-heart rate variability, SDNN-standard deviation of NN intervals, RMSSD – square root of the mean squared difference of successive NNs, TP-total power, VLF-very low frequency, LF-low frequency, HF-high frequency. Symbols: *P<0.05 vs wild-type. Table S2. Characterization of acetylcholine-induced currents in sinoatrial cells of wild-type, *Rgs6^−/−^*, and *Girk4^−/−^* mice. SAN cells were prepared from at least 3 different mice (6–8 weeks) per genotype. Parameters listed in the table were extracted from recordings of 11–15 cells per genotype. Steady-state (S-S) current was measured just prior to ACh removal. Desensitization was defined as: 100 * [(peak current - S-S current)/peak current]. Symbols: *P<0.05 vs wild-type, **P<0.01 vs wild-type, ***P<0.001 vs wild-type. Table S3. Heart rate variability parameters in conscious freely moving wild-type and *Rgs6^−/−^* mice. Wild-type (n = 4) and *Rgs6^−/−^* (n = 4) littermates (4–5 months old) were used. Abbreviations: HRV-heart rate variability, SDNN-standard deviation of NN intervals, RMSSD – square root of the mean squared difference of successive NNs, TP-total power, VLF-very low frequency, LF-low frequency, HF-high frequency. Symbols:*P<0.05 vs wild-type. Table S4. Heart rate variability parameters in human subjects with and without (wt) mutations in *Rgs6*. Abbreviations: SDNN – standard deviation of NN intervals, RMSSD – square root of the mean square difference of successive NN, SDSD – standard deviation of successive NN differences, SDANN – standard deviation of average NN intervals; pNN50 – the proportion of NN50 divided by total number of NNs, where NN50 is the number of pairs of successive NNs that differ by more than 50 ms; HRV – heart rate variability, TINN – triangular interpolation of NN intervals, VLF – very low frequency, LF – low frequency, HF – high frequency, VHF – very high frequency, nu – normalized units. Symbols: *P<0.05 (2μ **P<0.01 (3μ), ***P<0.0001 (6μ). Figure S1. Tachograms showing changes in RR intervals of baseline ECG recorded from all wild-type (black), *Rgs6^−/−^* (red), and *Girk4^−/−^* (green) isolated hearts used for the heart variability analysis. Figure S2. Poincare plots showing non-linear HRV analysis of beat-to-beat variation in RR duration of baseline ECG recorded from all wild-type (black), *Rgs6^−/−^* (red), and *Girk4^−/−^* (green) isolated hearts used for the heart variability analysis. 1000 consecutive data points was used for each graph.
(DOC)

Movie S1 **Time-lapse recordings of calcium flux in SAN from wild-type and** ***Rgs6^−/−^*** **mice.**
(AVI)
