# Supplementary material for: Essential Role of the m2R-RGS6-IKACh Pathway in Controlling Intrinsic Heart Rate Variability
Source: PLoS One. 2013 Oct 29;8(10):e76973. doi: 10.1371/journal.pone.0076973 (PMC3812209; doi:10.1371/journal.pone.0076973)
Supplement: File S1 — File includes Table S1–S4 and Figures S1–S2. Table S1. Heart rate variability parameters in isolated hearts of wild-type, Rgs6−/−, Girk4−/−, and Rgs6−/−:Girk4−/− double knockout mice. Hearts were isolated from 10–12 mice (8–16 weeks old) per genotype. Abbreviations: HRV-heart rate variability, SDNN-standard deviation of NN intervals, RMSSD – square root of the mean squared difference of successive NNs, TP-total power, VLF-very low frequency, LF-low frequency, HF-high frequency. Symbols: *P<0.05 vs wild-type. Table S2. Characterization of acetylcholine-induced currents in sinoatrial cells of wild-type, Rgs6−/−, and Girk4−/− mice. SAN cells were prepared from at least 3 different mice (6–8 weeks) per genotype. Parameters listed in the table were extracted from recordings of 11–15 cells per genotype. Steady-state (S-S) current was measured just prior to ACh removal. Desensitization was defined as: 100 * [(peak current - S-S current)/peak current]. Symbols: *P<0.05 vs wild-type, **P<0.01 vs wild-type, ***P<0.001 vs wild-type. Table S3. Heart rate variability parameters in conscious freely moving wild-type and Rgs6−/− mice. Wild-type (n = 4) and Rgs6−/− (n = 4) littermates (4–5 months old) were used. Abbreviations: HRV-heart rate variability, SDNN-standard deviation of NN intervals, RMSSD – square root of the mean squared difference of successive NNs, TP-total power, VLF-very low frequency, LF-low frequency, HF-high frequency. Symbols:*P<0.05 vs wild-type. Table S4. Heart rate variability parameters in human subjects with and without (wt) mutations in Rgs6. Abbreviations: SDNN – standard deviation of NN intervals, RMSSD – square root of the mean square difference of successive NN, SDSD – standard deviation of successive NN differences, SDANN – standard deviation of average NN intervals; pNN50 – the proportion of NN50 divided by total number of NNs, where NN50 is the number of pairs of successive NNs that differ by more than 50 ms; HRV – heart rate variability, TINN – tri [file pone.0076973.s001.doc]

**SUPPLEMENTAL MATERIALS**

**Table S1. Heart rate variability parameters in isolated hearts of wild-type, *Rgs6^-/-^,* *Girk4^-/-^,* and *Rgs6^-/-^:Girk4^-/-^*double knockout mice.**

| **HRV Parameter** | **Wild-type** | ***Rgs6^-/-^*** | ***Girk4^-/-^*** | ***Rgs6^-/-^:Girk4^-/-^*** |
| --- | --- | --- | --- | --- |
| ΔNN | 15.13±1.696 | 29.99±4.731* | 9.420±1.696* | 13.17±1.944 |
| SDNN, ms | 2.497±0.206 | 5.118±0.841* | 1.688±0.347* | 2.552±0.338 |
| RMSSD, ms | 1.318±0.287 | 3.875±1.107* | 0.573±0.188* | 0.757±0.165 |
| TP, ms^2^ | 5.162±0.870 | 28.52±8.356* | 2.285±0.710* | 4.767±1.203 |
| LF, ms^2^ | 0.479±0.166 | 4.501±1.942 | 0.263±0.157 | 0.187±0.075 |
| HF, ms^2^ | 0.536±0.171 | 6.259±3.493 | 0.118±0.051* | 0.177±0.066 |
| LF, nu | 49.86±3.797 | 48.60±5.449 | 61.25±7.019 | 48.44±3.582 |
| HF, nu | 50.14±3.797 | 51.40±5.449 | 38.85±7.017 | 51.56±3.582 |
| LF/HF | 1.126±0.168 | 1.437±0.536 | 2.754±0.756 | 1.046±0.178 |

Hearts were isolated from 10-12 mice (8-16 weeks old) per genotype. Abbreviations: HRV-heart rate variability, SDNN-standard deviation of NN intervals, RMSSD – square root of the mean squared difference of successive NNs, TP-total power, VLF-very low frequency, LF-low frequency, HF-high frequency. Symbols: *P<0.05 vs wild-type.

**Table S2. Characterization of acetylcholine-induced currents in sinoatrial cells of wild-type, *Rgs6^-/-^,* and *Girk4^-/-^*mice.**

| **Parameter** | **Wild-type** | ***Rgs6^-/-^*** | ***Girk4^-/-^*** | **ANOVA** |
| --- | --- | --- | --- | --- |
| Capacitance, pF | 65.2±5.1 | 52.7 ± 3.5 | 58.2 ± 4.4 | F_2,36_=1.9  *P*=0.17 |
| RMP, mV | -71.8 ± 0.4 | -73.7 ± 0.3* | -69.6 ± 0.7** | F_2,29_=15.5  *P*<0.0001 |
| Peak current density, pA | -35.1 ± 4.6 | -32.0 ± 3.0 | n.d. |  |
| S-S current density, pA | -26.0 ± 3.4 | -24.3 ± 3.4 | n.d. |  |
| Desensitization, % | 26.1 ± 2.2 | 23.3 ± 0.9 | n.d. |  |
| τ_act_, ms | 36.3 ± 2.1 | 43.2 ± 2.8 | n.d. |  |
| τ_deact_, ms | 512 ± 73 | 3020 ± 239*** | n.d. |  |

SAN cells were prepared from at least 3 different mice (6-8 weeks) per genotype. Parameters listed in the table were extracted from recordings of 11-15 cells per genotype. Steady-state (S-S) current was measured just prior to ACh removal. Desensitization was defined as: 100 * [(peak current - S-S current)/peak current]. Symbols: *P<0.05 vs wild-type, **P<0.01 vs wild-type, ***P<0.001 vs wild-type.

**Table S3. Heart rate variability parameters in conscious freely moving wild-type and *Rgs6^-/-^*mice.**

| **HRV Parameter** | **Wild-type** | ***Rgs6^-/-^*** |
| --- | --- | --- |
| ΔNN, ms | 95.81±11.42 | 333.1±104.4* |
| SDNN, ms | 10.71±2.828 | 20.25±0.592* |
| RMSSD, ms | 4.876±1.552 | 8.441±2.074 |
| NN50 | 2.250±0.854 | 19.75±14.17 |
| NN50, % | 0.044±0.018 | 0.413±0.295 |
| TP, ms^2^ | 32.31±15.05 | 72.66±27.37 |
| LF, ms^2^ | 11.30±5.405 | 21.07±9.631 |
| HF, ms^2^ | 9.088±4.616 | 22.93±11.00 |
| LF, nu | 58.68±2.810 | 50.71±2.903 |
| HF, nu | 41.32±2.810 | 49.29±2.903 |
| LF/HF | 1.526±0.210 | 1.052±0.131 |

Wild-type (n=4) and *Rgs6^-/-^* (n=4) littermates (4-5 months old) were used. Abbreviations: HRV-heart rate variability, SDNN-standard deviation of NN intervals, RMSSD – square root of the mean squared difference of successive NNs, TP-total power, VLF-very low frequency, LF-low frequency, HF-high frequency. Symbols:*P<0.05 vs wild-type.

**Table S4. Heart rate variability parameters in human subjects with and without (wt) mutations in *Rgs6.***

| **Value** | **wt** | **p.A461V** | **p. A270S** | **p. L73F** | **p.V13LfsX11** |
| --- | --- | --- | --- | --- | --- |
| SDNN, ms | 135.9±11.58 | 127.1 | 242.3* | 87.0 | 185.3 |
| SDNN Day, ms | 90.38±9.211 | 103.9 | 150.7 | 89.9 | 146.3 |
| SDNN Night, ms | 106.4±15.37 | 74.8 | 134.9 | 56.6 | 127.0 |
| RMSSD, ms | 34.78±4.381 | 25.5 | 41.6 | 24.6 | 74.9* |
| RMSSD Day, ms | 27.07±2.522 | 23.1 | 18.7 | 22.9 | 45.7* |
| RMSSD Night, ms | 34.86±4.184 | 29.1 | 69.5 | 26.6 | 110.4* |
| SDSD, ms | 28.12±4.126 | 19.5 | 35.5 | 17.4 | 62.3* |
| SDSD Day, ms | 21.19±2.156 | 17.2 | 13.6 | 16.0 | 36.0* |
| SDSD Night, ms | 33.02±7.235 | 22.7 | 55.9 | 18.9 | 87.9* |
| SDANN, ms | 127.8±10.92 | 119.9 | 223.0* | 73.6 | 156.8 |
| SDANN Day, ms | 81.80±9.192 | 99.3 | 130.2 | 74.9 | 117.8 |
| SDANN Night, ms | 83.85±13.55 | 63.1 | 73.8 | 44.5 | 76.1 |
| pNN50, % | 5.855±1.276 | 2.0 | 7.7 | 5.2 | 20.6* |
| pNN50 Day, % | 3.645±1.034 | 1.9 | 1.1 | 2.2 | 12.4* |
| pNN50 Night, % | 10.01±3.061 | 2.3 | 22.6 | 3.3 | 35.9* |
| HRV Index | 16.14±1.425 | 14.6 | 18.2 | 10.4 | 25.9* |
| TINN | 462.4±52.46 | 531.3 | 390.6 | 296.9 | 703.1 |
| VLF Day, ms^2^ | 557.0±125.0 | 690.6733 | 485.3010 | 637.0715 | 1363.8460 |
| LF Day, ms^2^ | 643.8±164.6 | 633.1502 | 677.6600 | 418.0344 | 2756.6140** |
| HF Day, ms^2^ | 204.5±56.83 | 217.1760 | 78.8571 | 120.4302 | 692.4813* |
| VHF Day, ms^2^ | 53.63±8.929 | 48.2465 | 24.2552 | 35.0232 | 82.0104 |
| LF Day, nu | 68.66±3.450 | 53.9993 | 79.2824 | 73.2614 | 75.5323 |
| HF Day, nu | 22.34±2.457 | 34.0519 | 12.7847 | 20.1203 | 20.8732 |
| LF/HF Day | 4.177±0.659 | 2.0867 | 7.6798* | 3.9452 | 4.0783 |
| VLF Night, ms^2^ | 1468±453.3 | 546.2554 | 4591.7630* | 427.5419 | 2088.2440 |
| LF Night, ms^2^ | 1629±661.2 | 373.0122 | 5094.5310 | 378.8511 | 3815.9220 |
| HF Night, ms^2^ | 681.7±263.0 | 200.8354 | 1033.5200 | 285.2979 | 2564.7000* |
| VHF Night, ms^2^ | 92.07±37.72 | 63.1383 | 46.1770 | 43.0239 | 1084.1720*** |
| LF Night, nu | 61.57±4.728 | 47.2481 | 78.3024 | 53.0660 | 52.6843 |
| HF Night, nu | 31.83±4.143 | 40.3206 | 20.9231 | 38.2992 | 35.1637 |
| LF/HF Night | 2.774±0.372 | 2.6889 | 5.2513 | 1.6117 | 1.6181 |

Abbreviations: SDNN – standard deviation of NN intervals, RMSSD – square root of the mean square difference of successive NN, SDSD – standard deviation of successive NN differences, SDANN – standard deviation of average NN intervals; pNN50 – the proportion of NN50 divided by total number of NNs, where NN50 is the number of pairs of successive NNs that differ by more than 50ms; HRV – heart rate variability, TINN – triangular interpolation of NN intervals, VLF – very low frequency, LF – low frequency, HF – high frequency, VHF – very high frequency, nu – normalized units. Symbols: *P<0.05 (2σ), **P<0.01 (3σ), ***P<0.0001 (6σ).

**Figure S1.** Tachograms showing changes in RR intervals of baseline ECG recorded from all wild-type (black), *Rgs6^–/–^* (red), and *Girk4^–/–^* (green) isolated hearts used for the heart variability analysis.

**Figure S2.** Poincare plots showing non-linear HRV analysis of beat-to-beat variation in RR duration of baseline ECG recorded from all wild-type (black), *Rgs6^–/–^* (red), and *Girk4^–/–^* (green) isolated hearts used for the heart variability analysis. 1000 consecutive data points was used for each graph.
